# Maxillofacial free flap surgery outcomes in critical care: a single-center investigation looking for clues to improvement

**DOI:** 10.1186/s13741-022-00244-5

**Published:** 2022-03-10

**Authors:** Bruno Denis, Claire Gourbeix, Marine Coninckx, Jean-Philippe Foy, Chloé Bertolus, Jean-Michel Constantin, Vincent Degos

**Affiliations:** 1 Department of Anesthesia and Intensive Care, Pitié-Salpêtrière Hospital, Boulevard de l’Hôpital 47-83, 75013 Paris, France; 2 Intensive Care Unit, Saint-Luc Hospital, Université Catholique de Louvain, Avenue Hippocrate 10, 1200 Brussels, Belgium; 3 Maxillofacial Surgery Unit, Pitié-Salpêtrière Hospital, Boulevard de l’Hôpital 47-83, 75013 Paris, France

**Keywords:** Perioperative medicine, Intensive care, Critical care, Free flap, Maxillofacial surgery, Head and neck reconstruction, Microvascular surgery, Postoperative complications, Perioperative improvement, Prehabilitation

## Abstract

**Background:**

Maxillofacial surgery for free flap reconstructions is associated with many complications due to technical complexity and comorbidity of patients. With a focus on critical care, the authors studied the impact of complications to highlight predictors of poor postoperative outcomes in order to implement optimization protocols.

**Methods:**

This case-control study analyzed the relationship between perioperative variables and postoperative medical and surgical complications of patients who underwent head and neck surgery using fibular and forearm free flaps. The primary objective was the incidence of prolonged intensive care unit (ICU) length of stay (LOS). Secondary objectives were the incidence of ICU readmissions, postoperative infections, and 1-year mortality. A univariable logistic regression model was used. A study of mortality was performed with survival analysis. Regarding our primary objective, we performed a Benjamini-Hochberg procedure and a multivariable Poisson regression with defined variables of interest.

**Results:**

The data of 118 hospital stays were included. Prolonged ICU LOS was observed in 47% of cases and was associated with chronic obstructive pulmonary disease, pneumopathies, intraoperative blood transfusion, and surgical duration. Medical and surgical complications were associated with prolonged ICU LOS. After the Benjamini-Hochberg procedure, infectious complications, complications, major complications, total number of pneumopathies, and operative time remained significant. At least one complication was experienced by 71% of patients during the hospitalization, and 33% of patients suffered from major complications. Infectious complications were the most common (40% of patients) and were mainly caused by pneumonia (25% of patients); these complications were associated with low preoperative hemoglobin level, intraoperative blood transfusion, accumulation of reversible cardiovascular risk factors, chronic alcohol consumption, and duration of surgery. Pneumonia was specifically associated with chronic obstructive pulmonary disease. The ICU readmission rate was 10% and was associated with lower preoperative hemoglobin level, pneumopathies, surgical duration, and use of a fibular flap. The 1-year mortality was 12%, and the survival analysis showed no association with prolonged ICU LOS. Poisson regression showed that ICU LOS was prolonged by smoking history, lower preoperative hemoglobin level, intraoperative blood transfusion, major complication, and pneumopathies.

**Conclusions:**

Practices such as blood management and respiratory prehabilitation could be beneficial and should be evaluated as a part of global improvement strategies.

## Background

The aim of the use of maxillofacial free flaps (FF) for head and neck reconstruction is to limit functional (swallowing, mastication and speech) and esthetic consequences of surgical interventions. Therefore, they are key for the rehabilitation process (Zaghi et al., 2014). However, surgical complexity and comorbidity (especially in cancer contexts) result in recurrent postoperative complications which can undermine the expected benefits. Complications are associated with increased hospital LOS (Eskander et al., 2018; Lahtinen et al., 2018), readmission (Carniol et al., 2017), and mortality (McMahon et al., 2017). The implementation of specific perioperative optimization protocols such as alcohol cessation (Kaka et al., 2017) or adapted postoperative care (Arshad et al., 2014; Varadarajan et al., 2017) can improve outcomes and reduce hospitalization costs. Enhanced recovery after surgery (ERAS) protocols applied to maxillofacial FF seem also to improve the recovery in intensive care units (ICU) (Bertelsen et al., 2020), but few studies focus on critical care. Moreover, due to heterogeneity in patient characteristics, surgical techniques, and perioperative management, many disparities in postoperative evolution are seen in the literature (Marsh et al., 2009; Whitaker et al., 2007). The lack of agreement on the definition of complications also leads to report inconsistencies (Perisanidis et al., 2012). All these make the comparison of practices difficult.

In this framework, we decided to carry out our own investigation to get more information about the outcomes of our current practices. We wanted to find clues that could lead to new protocols to improve patient care. We focused on critical care evolution due to the lack of data available in this setting, where patient management is particularly challenging and requires many human and material resources. The primary objective was to highlight the risk factors of prolonged ICU LOS, and the secondary objective was to study ICU readmission. We also analyzed the risk factors of postoperative infections, which are frequent complication and mortality.

## Methods

### Design, setting, and participants

A single-center case-control study was conducted at the tertiary care center Pitié-Salpêtrière in Paris, France. We collected the perioperative data of all patients who underwent head and neck reconstruction by fibular and forearm FF between January 2018 and December 2019. We focused on these two types of flaps to improve surgical homogeneity, and multiple flaps were excluded. Cancer, trauma, and osteoradionecrosis were included as indications. Our current medical practices were already in place during this two-year period, which permitted 1 year follow-up for the survival analysis.

### Postoperative management in our institution

Reconstructions with antebrachial FF are mostly performed after soft tissue resection, while fibular FFs are used when the resection includes the bone. After surgery, patients are transferred to the post-anesthesia care unit (PACU) for one night, where similar surveillance to intensive care is applied. Attention is paid to hemodynamic and intermittent positive pressure ventilation is used for respiratory optimization (Chiumello et al., 2011). A cutoff of 10 g/dL of hemoglobinemia is fixed for perioperative blood transfusion. Later on, patients are relocated either to the surgical ward or the intensive care unit, depending on their comorbidities, course of surgery, and PACU evolution. Antibiotic prophylaxis with amoxicillin-clavulanic acid (or clindamycin associated with gentamicin if penicillin allergy is reported) is applied for 48 h if there is no current operative site infection (Veve et al., 2017). The non-specific management consists of acute pain management, early thromboprophylaxis, systematic wound care, and early nutrition in the continuity of ERAS guidelines (Dort et al., 2017).

### Variables

Preoperative variables consisted of patient characteristics and comorbidities collected from the anesthesiologic records. A patient’s comorbidity was defined as any past medical history reported by an attending physician. Intraoperative data included the type of flap performed, ischemia and operative times, quantity of intravenous fluids administered, and need for blood transfusion. Postoperative complication was defined as any reported deviation from the normal postoperative course and was considered major if the complication resulted in a return to the operating room or if initiation or prolongation of organ support was needed. Initial PACU stays and eventual ICU readmissions were included in the total ICU LOS count. Complications were followed until the end of the hospital stay and we performed a 1-year follow-up for the mortality.

### Data sources

Data were collected from both paper and electronic medical files. Preoperative anesthesiological evaluation, intraoperative data, and surgical ward evolution were documented on paper files. Postoperative ICU records, surgical follow-up, and laboratory analyses were recorded in electronic files (Metavision, iMDsoft; Orbis, AFGA).

### Study size

The number of patients who underwent maxillofacial FF surgeries during the defined period determined the sample size.

### Quantitative variables

Regarding our primary objective, a LOS cutoff of 5 days was initially set after reviewing the median duration expected for ICU LOS following FF surgery in our institution. A LOS of 5 days or more was considered prolonged. Patients with a body mass index (BMI) lower than 18.5 kg/m^−2^ were considered malnourished (National Institute for Health and Clinical Excellence (NICE), 2017). Other continuous variables were not grouped.

### Statistical analysis

Statistical analysis was performed using Rstudio (v. 1.4.1106), Pvalue.io (Medistica., 2019. https://www.pvalue.io), and JPM Pro (v. 16.0.0) software. A *P*-value less than 0.05 was considered statistically significant. Quantitative variables were compared with a Wilcoxon test and qualitative values were compared with a Fisher or *χ*^2^ test. A continuity correction was applied when a group contained less than five elements. Covariates were compared for each outcome using a univariable logistic regression model. The log-rank test was used for survival analysis. Regarding our primary objective, a Benjamini-Hochberg procedure with a 5% false discovery rate was performed following multiple testing, and we used a multivariable Poisson regression with variables of interest identified through our study to consider the ICU LOS a continuous variable.

## Results

### Participants and flow diagram

**Fig. 1:**
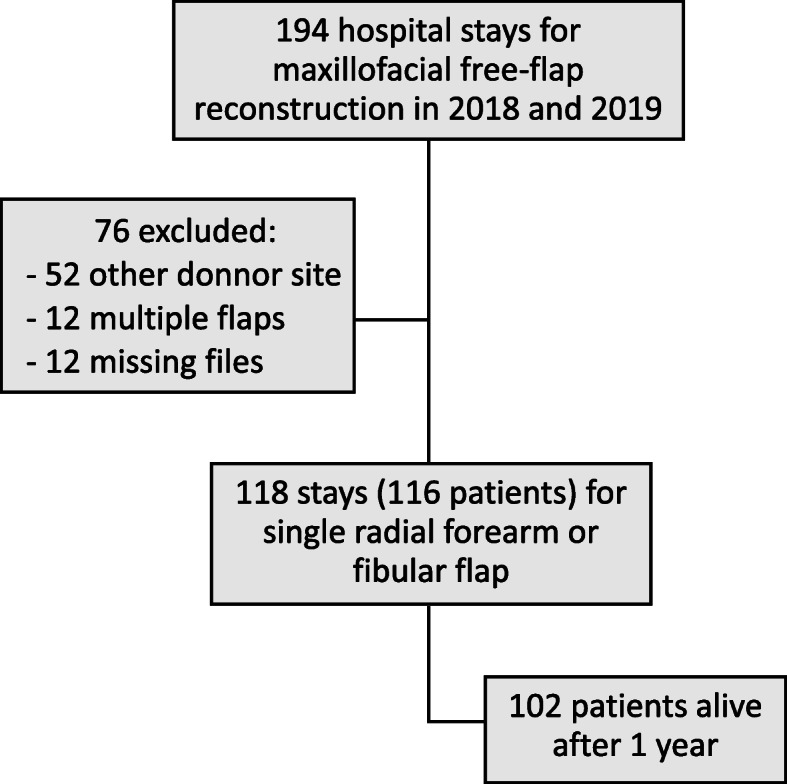
Flow chart

One hundred ninety-four hospital stays for maxillofacial reconstructions were recorded during the 2-year period. Seventy-six were excluded: 52 were not fibular nor forearm flaps, 12 were multiple flaps, and 12 files were missing. Therefore, 118 hospital stays were included and corresponded to 116 patients. One hundred two of them were alive after 1 year (Fig. 1).

### Patient characteristics

Patient characteristics and perioperative data are reported in Tables 1 and 2. The average age was 60 years (SD = 15.9) and 61% (*n* = 72) of the enrolled patients were male. The American Society of Anesthesiologists (ASA) physical status classification system was applied to the patients: 64% (*n* = 75) were class II, and 24% (*n* = 28) were class III. Malnutrition status was assigned if the patient’s BMI was less than 18.5 kg m^−2^ (8%, *n* = 9). The most common comorbidity was cigarette smoking; 65% (*n* = 77) of patients had a history of smoking and 35% (*n* = 41) were active smokers. Other common comorbidities were regular alcohol consumption (52%, *n* = 61), arterial hypertension (42%, *n* = 49), cardiovascular disease (25%, *n* = 30), and chronic obstructive pulmonary disease (COPD) (15%, *n* = 18). Fibular flaps were used in 53% (*n* = 63) of the reconstructions. The main indication for surgery was malignant tumor resection (82%, *n* = 97), of which squamous cell carcinoma was the most frequent (74%, *n* = 87). Delayed reconstructions (i.e., flap surgeries that took place after the initial tumor resection) were also performed (13%, *n* = 15). The mean operative duration was 7 h (SD = 1.9). Intraoperative blood transfusion was required in 63% (*n* = 74) of the procedures. The mean ICU LOS was 6 days (SD = 4.9), and the mean hospital LOS was 21 days (SD = 16.4).

**Table 1:** Patients characteristics and comorbidities

***Variables***	***Value***
**Age** (years) *(n = 118)*	59.5 ± 15.9
**Sex** *(n = 118)*	
Male	72 (61.0)
Female	46 (39.0)
**BMI** (kg m^−2^) *(n = 114)*	23.9 ± 4.4
< 18.5	9 (7.9)
≥ 30	11 (9.7)
**Hypertension** *(n = 117)*	49 (41.9)
**Dyslipidemia** *(n = 117)*	23 (19.7)
**Diabetes** *(n = 117)*	10 (8.6)
**Addictions**	
Active smoking *(n = 118)*	41 (34.8)
Smoking history *(n = 118)*	77 (65.3)
Alcohol *(n = 117)*	61 (52.1)
Cannabis *(n = 115)*	10 (8.7)
**Cardiovascular disease** *(n = 118)*	30 (25.4)
Heart disease	19 (16.1)
Occlusive peripheral arterial disease	7 (5.9)
Carotid occlusive disease	11 (9.3)
**COPD** *(n = 118)*	18 (15.3)
**Chronic renal failure** *(n = 118)*	9 (7.6)
**Liver disease** *(n = 118)*	7 (5.9)
**Stroke** *(n = 118)*	4 (3.4)
**Charlson Comorbidity Index** *(n = 117)*	4.1 ± 2.1
**ASA classification** *(n = 118)*	
I	15 (12.7)
II	75 (63.6)
III	28 (23.7)

Categorical variables are presented as *n* (%) and continuous variables as mean ± SD

Due to missing data, the number of patients included is presented as (*n* =) after each variable

*ASA* American Society of Anesthesiologists, *BMI* body mass index, *COPD* chronic obstructive pulmonary disease

**Table 2:** Perioperative data

***Variables***	***Value***
**Surgical indication** *(n = 118)*	
Primary tumoral resection	88 (74.6)
Delayed reconstruction after tumoral resection	15 (12.7)
Osteoradionecrosis	9 (7.6)
Trauma	3 (2.5)
Others	3 (2.5)
**Resection site** (n = 98*)	
Bone	54 (55.1)
Soft tissue	26 (26.5)
Broad resection	16 (16.3)
Others	2 (2.0)
**Cancer context** *(n = 118)*	97 (82.2)
Squamous cell carcinoma	87 (73.7)
**Past head and neck radiotherapy** *(n = 118)*	27 (22.9)
**Past chemotherapy** *(n = 114)*	22 (19.3)
**Past head and neck FF failure** *(n = 118)*	5 (4.2)
**Preoperative hemoglobinemia** (g/dL) *(n = 76)*	13.0 ± 1.6
**Flap** *(n = 118)*	
Radial forearm	55 (46.6)
Fibula	63 (53.4)
**Ischemia time** (min) *(n = 98)*	56.3 ± 29.9
**Operative time** (h) *(n = 114)*	7.1 ± 1.9
**Intraoperative IV fluids** (mL.kg^−1^.h^−1^) *(n = 108)*	9.8 ± 3.3
**Intraoperative blood transfusion** *(n = 118)*	74 (62.8)
**ICU LOS** (days)	5.8 ± 4.9
Total LOS (days)	21.2 ± 16.4

Categorical variables presented as *n* (%) and continuous variables as mean ± SD

*FF* free flap, *ICU* intensive care unit, *IV* intravenous, *LOS* length of stay

*Delayed reconstructions for any indication were excluded from the patient count

### Postoperative complications

**Fig. 2:**
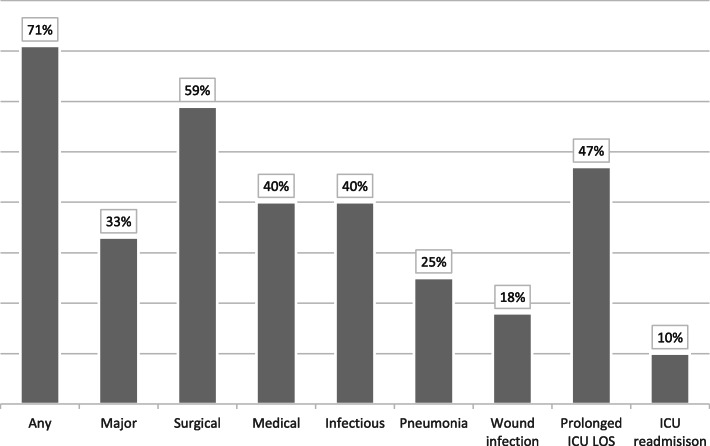
Postoperative complications. ICU, intensive care unit; LOS, length of stay. Infectious complication includes pneumonia and wound infection; wound infection concerns both donor and recipient sites

Postoperative complications (Table 3, Fig. 2) were reported in 71% (*n* = 84) of hospitalizations and were major in 33% (*n* = 39) of the cases. Surgical complications were most frequent (59%, *n* = 69), mainly due to wound healing problems at donor and recipient sites (28%, *n* = 33). Total flap necrosis occurred in 7% (*n* = 8) of cases. Medical complications were observed in 40% (*n* = 47) of patients; of the complications, pneumonia was the most frequent (25%, *n* = 30). Infectious complications occurred in 40% (*n* = 47) of cases, including pneumonia, wound infection (18%, *n* = 21), and extra-pulmonary sepsis (4%, *n* = 5). ICU LOS was prolonged in 47% (*n* = 55) of cases. Reintervention was needed in 31% (*n* = 36) of cases, and of them, 10% (*n* = 12) required ICU readmission. Only one death was reported during hospitalization. The 1-year mortality was 12% (*n* = 14) and was mainly the result of tumor progression (79%, *n* = 11), followed by respiratory complications (21%, *n* = 3). Thus, every death during the first postoperative year occurred in cases with cancer, and the mortality for this specific population was 14% (*n* = 14).

**Table 3:** Complications *(n = 118)*

***Variables***	***Value***
**Surgical**	69 (58.5)
Wound healing	33 (28.0)
Wound infection	21 (17.8)
Hemorrhage	17 (14.4)
Partial necrosis	13 (11.0)
Orostoma	11 (9.3)
Venous thrombosis	10 (8.5)
Postoperative tracheotomy	8 (6.8)
Total necrosis	8 (6.8)
Arterial thrombosis	5 (4.2)
**Surgical reintervention**	36 (30.5)
**Medical**	47 (39.8)
Pneumonia	30 (25.4)
Metabolic	8 (6.8)
Neurological	6 (5.1)
Cardiovascular	5 (4.2)
Extra-pulmonary sepsis	5 (4.2)
Atrial fibrillation	4 (3.4)
Acute lung edema	3 (2.5)
Abdominal	3 (2.5)
Respiratory (pneumonia excluded)	2 (1.7)
Pulmonary embolism	2 (1.7)
**Infectious**	47 (39.8)
**Any complication**	84 (71.2)
**Major complication**	39 (33.1)
**Prolonged ICU LOS**	55 (46.6)
**ICU readmission**	12 (10.2)
**Mortality**	
During hospitalization	1 (0.9)
1 year *(n = 116)*	14 (12.1)

Categorical variables are presented as *n* (%) and continuous variables as mean ± SD

*ICU* intensive care unit, *LOS* length of stay

Due to missing data, the number of patients included is presented as (*n* =) after each variable

Wound healing and wound infection problems include both donor and recipient sites while other surgical complications were observed only at the recipient site

Metabolic complications include significant ionic and glycemic disturbances

### Statistical analysis

Following univariate analysis, prolonged ICU LOS (equal to or greater than five days) (Table 4) was associated with COPD (OR = 3.42, *p* = 0.024), total number of pneumopathies (OR = 3.78, *p* = 0.002), intraoperative blood transfusion (OR = 2.21, *p* = 0.044), and increased surgical duration (OR = 1.37, *p* = 0.006). The occurrence of complication (OR = 4.89, *p* = 0.001), including major (OR = 3.46, *p* = 0.002) and infectious (OR = 4.11, *p* = < 0.001), was also associated with prolonged ICU LOS. When the Benjamini-Hochberg procedure was performed, the occurrence of all complications (*q* = 0.003), infectious complications (*q* = 0.002), major complications (*q* = 0.005), total number of pneumopathies (*q* = 0.006), and operative time (*q* = 0.008) remained significant. ICU readmission (Table 5) was associated with low initial hemoglobin level (OR = 0.56, *p* = 0.029), the number of pneumopathies (OR = 2.40, *p* = 0.033), surgical duration (OR = 1.46, *p* = 0.041), and the use of a fibular flap (OR = 11.42, *p* = 0.022). Infectious complications (Table 6) were associated with low preoperative hemoglobin level (OR = 0.65, *p* = 0.012), intraoperative blood transfusion (OR = 2.39, *p* = 0.034), accumulation of reversible cardiovascular risk factors (OR = 1.55, *p* = 0.025), chronic alcohol consumption (OR = 3.02, *p* = 0.005), and surgical duration (OR = 1.40, *p* = 0.004). Pneumonia was specifically associated with COPD (OR = 3.01, *p* = 0.039). Survival analysis showed no association between the one-year mortality and prolonged ICU LOS (*p* = 0.16). We performed a Poisson regression (Table 7) with variables related to respiratory pathologies, blood management, complications, and operative time. Smoking history (*p* ≤ 0.001), pneumopathies (*p* ≤ 0.001), low preoperative hemoglobin level (*p* ≤ 0.001), intraoperative blood transfusion (*p* = 0.036), and major complications (*p* ≤ 0.001) significantly prolonged ICU LOS.

**Table 4:** Univariable analysis of predictors of prolonged ICU LOS

***Variables***	Univariable OR (95% CI)	Univariable ***p***-value
**Age** (years)	1.01 (0.98–1.03)	0.501
**Sex**	1.77 (0.84–3.78)	0.137
**BMI** (kg m^−2^)	1.02 (0.94–1.11)	0.665
< 18.5 kg m^−2^	0.69 (0.14–2.78)	0.618
**Smoking history**	1.37 (0.63–3.03)	0.427
**Alcohol**	1.63 (0.78–3.46)	0.196
**Total reversible CV risk factors**	1.10 (0.76–1.59)	0.619
**CV disease**	1.32 (0.57–3.06)	0.509
**COPD**	**3.41 (1.22–0.50)**	**0.024**
**Chronic renal failure**	0.38 (0.05–1.64)	0.237
**Liver disease**	1.96 (0.41–0.32)	0.395
**Stroke**	1.43 (0.17–2.22)	0.728
**Charlson Comorbidity Index**	1.08 (0.91–1.30)	0.387
**ASA classification**		
II	2.11 (0.63–8.39)	0.248
III	2.73 (0.69–2.38)	0.166
**Surgical indication**		
Primary tumoral resection	0.35 (0.07–1.40)	0.151
Delayed reconstruction	0.25 (0.04–1.36)	0.121
Osteoradionecrosis	0.35 (0.07–1.40)	0.151
Trauma	0.25 (0.01–3.67)	0.327
Others	0.25 (0.01–3.67)	0.327
**Cancer context**	0.60 (0.21–1.69)	0.330
**Past head and neck radiotherapy**	1.15 (0.47–2.73)	0.758
**Past head and neck FF failure**	3.00 (0.36–1.12)	0.318
**Preoperative hemoglobin level** (g/dL)	0.87 (0.67–1.09)	0.270
**Fibula flap**	1.72 (0.82–3.66)	0.152
**Ischemia time** (min)	1.00 (0.99–1.02)	0.704
**Operative time** (h)	**1.37 (1.10–1.73)**	0.006*
**Intraoperative IV fluids** (mL kg^−1^ h^−1^)	0.98 (0.87–1.10)	0.706
**Intraoperative blood transfusion**	**2.26 (1.04–5.11)**	**0.044**
**Any complication**	**4.89 (1.94–4.19)**	0.001*
**Major complication**	**3.46 (1.57–7.84)**	0.002*
**Infectious complication**	**4.11 (1.90–9.16)**	< 0.001*
**Total pneumopathies**	**3.78 (1.74–9.18)**	0.002*

Categorical variables are presented as *n* (%) and continuous variables as mean ± SD. Bold values are significant. *p*-values marked with [*] remain significant after a Benjamini-Hochberg procedure with a false discovery rate of 5%

*BMI* body mass index, *COPD* chronic obstructive pulmonary disease, *CV* cardiovascular, *FF* free flap, *ICU* intensive care unit, *IV* intravenous, *LOS* length of stay

Delayed reconstructions are flap surgeries that take place time after an initial resection

Reversible cardiovascular risk factors include obesity, hypertension, dyslipidemia, diabetes, and active smoking

**Table 5:** Univariable analysis of predictors of ICU readmission

***Variables***	Univariable OR (95% CI)	Univariable ***p***-value
**Age** (years)	1.03 (0.99–1.08)	0.130
**Sex**	1.16 (0.32–3.88)	0.810
**BMI** (kg m^−2^)	1.01 (0.87–1.15)	0.927
< 18.5	–	
**Smoking history**	1.09 (0.32–4.30)	0.896
**Alcohol**	2.00 (0.59–7.87)	0.281
**Total reversible CV risk factors**	1.73 (0.97–3.17)	0.065
**CV disease**	0.96 (0.20–3.50)	0.957
**COPD**	0.47 (0.02–2.67)	0.484
**Chronic renal failure**	1.10 (0.06–6.88)	0.930
**Liver disease**	–	
**Stroke**	3.09 (0.15–26.63)	0.346
**Charlson Comorbidity Index**	1.05 (0.79–1.41)	0.740
**ASA classification**		
II	–	
III	–	
**Surgical indication**		
Delayed reconstruction	–	
Osteoradionecrosis	–	
Trauma	–	
Other	–	
**Cancer context**	0.87 (0.20–6.04)	0.868
**Past head and neck radiotherapy**	0.67 (0.10–2.75)	0.616
**Past head and neck FF failure**	–	
**Preoperative hemoglobin level** (g/dL)	**0.56 (0.32**–**0.92)**	**0.029**
**Fibula flap**	**11.21 (2.07**–**208.60)**	**0.023**
**Ischemia time** (min)	1.02 (1.00–1.04)	0.048
**Operative time** (h)	**1.46 (1.03**–**2.15)**	**0.041**
**Intraoperative IV fluids** (mL.kg^−1^.h^−1^)	1.05 (0.86–1.25)	0.632
**Total pneumopathies**	**2.40 (1.04**–**5.59)**	**0.033**

Categorical variables are presented as *n* (%) and continuous variables as mean ± SD. Bold values are significant

*BMI* body mass index, *COPD* chronic obstructive pulmonary disease, *CV* cardiovascular, *FF* free flap, *ICU* intensive care unit, *IV* intravenous, *LOS* length of stay

Reversible cardiovascular risk factors include obesity, hypertension, dyslipidemia, diabetes, and active smoking

**Table 6:** Univariable analysis of predictors of infectious complications

***Variables***	Univariable OR (95% CI)	Univariable ***p***-value
**Age** (years)	1.02 (1.00–1.05)	0.071
**Sex**	0.71 (0.32–1.51)	0.371
**BMI** (kg m^−2^)	0.97 (0.89–1.06)	0.499
< 18.5	2.03 (0.51–8.63)	0.312
**Smoking history**	2.01 (0.91–4.64)	0.090
**Alcohol**	**3.02 (1.41–6.69)**	**0.005**
**Total reversible CV risk factors**	**1.55 (1.06–2.30)**	**0.025**
**CV disease**	0.84 (0.35–1.95)	0.682
**COPD**	2.13 (0.77–6.04)	0.144
**Chronic renal failure**	0.74 (0.15–2.96)	0.680
**Liver disease**	0.24 (0.01–1.44)	0.188
**Stroke**	1.53 (0.18–13.15)	0.675
**Charlson Comorbidity Index**	1.13 (0.95–1.36)	0.184
**ASA classification**		
II	1.71 (0.51–6.83)	0.406
III	2.29 (0.58–10.37)	0.252
**Surgical indication**		
Delayed reconstruction	0.63 (0.11–3.51)	0.587
Osteoradionecrosis	0.87 (0.21–3.70)	0.838
Trauma	0.63 (0.02–9.16)	0.736
Others	0.63 (0.02–9.16)	0.736
**Cancer context**	1.76 (0.60–5.88)	0.323
**Past head and neck radiotherapy**	0.84 (0.34–2.02)	0.705
**Past head and neck FF failure**	7.50 (0.78–101.01)	0.092
**Preoperative hemoglobin level** (g/dL)	**0.65 (0.45–0.90)**	**0.012**
**Fibula flap**	1.52 (0.72–3.23)	0.274
**Ischemia time** (min)	1.01 (0.99–1.02)	0.270
**Operative time** (h)	**1.40 (1.12–1.77)**	**0.004**
**Intraoperative IV fluids** (mL.kg^−1^.h^−1^)	1.03 (0.91–1.16)	0.662
**Intraoperative blood transfusion**	**2.39 (1.09–5.50)**	**0.034**

Categorical variables are presented as *n* (%) and continuous variables as mean ± SD. Bold values are significant

*BMI* body mass index, *COPD* chronic obstructive pulmonary disease, *CV* cardiovascular, *FF* free flap, *ICU* intensive care unit, *IV* intravenous, *LOS* length of stay

Reversible cardiovascular risk factors include obesity, hypertension, dyslipidemia, diabetes, and active smoking

**Table 7:** Multivariable Poisson regression analysis of prolonged ICU LOS (*n* = 72)

***Variables***	Rate ratio	***p***-value
**Smoking history**	**1.259**	**< 0.001**
**COPD**	0.987	0.834
**Preoperative hemoglobin level**	**0.875**	**< 0.001**
**Operative time**	0.952	0.111
**Intraoperative blood transfusion**	**1.138**	**0.036**
**Major complication**	**1.281**	**< 0.001**
**Infectious complication**	0.941	0.376
**Total pneumopathies**	**1.358**	**< 0.001**

Bold values are significant

*COPD* chronic obstructive pulmonary disease, *ICU* intensive care unit, *LOS* length of stay

## Discussion

A 2009 study conducted at our institution suggested a surgical complication rate of up to 56% (Chaine et al., 2009). Large cohort studies also reported comparable incidences of major postoperative complications and pneumonia (McMahon et al., 2017; Patel et al., 2010) and showed an association between these complications and prolonged hospital LOS and hospital readmission (Eskander et al., 2018); however, the impact of these findings on critical care has not yet been described. We investigated the causes of prolonged ICU LOS, ICU readmissions, and infectious complications to demonstrate their influence on perioperative medicine. All complications were associated with prolonged ICU LOS. Medical care in this setting requires many resources and the benefits of optimization protocols are evident for both patients and caregivers.

We found multiple associations between low preoperative hemoglobin level, blood transfusion, and poor postoperative outcomes. Morbidity due to perioperative blood product administration is widely demonstrated and described in head and neck FF reconstructions (Danan et al., 2015). Among these specific surgeries, anemia is also associated with postoperative complications (Mlodinow et al., 2013), and transfusion is identified as a risk factor of infections (Von Doersten et al., 1992) and poor prognosis among oncology patients (Szakmany et al., 2006). A transfusion cutoff has yet to be defined, but current evidence suggests the benefit of restrictive policies (Puram et al., 2015; Rossmiller et al., 2010). In some cases, blood transfusions are the consequence and not the cause of complications, especially in case of hemorrhage. These findings highlight the potential benefits of improved patient blood management.

The results of our study suggest that respiratory optimization could be an area of further research. Smoking is a major risk factor for both COPD and head and neck cancers; it was the main comorbidity in our population and was associated with prolonged ICU LOS in the Poisson regression. Smoking is associated with postoperative surgical complications (Garip et al., 2021; Clark et al., 2007) and reinterventions (Crippen et al., 2019). We found that COPD was associated with prolonged ICU LOS and pneumonia in our univariate analysis. Smoking is described as a risk factor for prolonged operative time (Lindeborg et al., 2020), which on its own was recurrently associated with complications. Finally, the number of pneumopathies was associated with prolonged ICU LOS and ICU readmission. Postoperative physical therapy is effective in reducing pulmonary complications (Dort et al., 2017) and is applied to our patients in addition to systematic positive pressure ventilation for at least 48 h postoperatively (Chiumello et al., 2011). Further implementations, such as respiratory prehabilitation, should be considered.

The perioperative mortality of FF surgeries is relatively low. An average 1-month mortality of 1.2% has recently been reported (Chicco et al., 2021). The only death that occurred during hospitalization in our study was due to hypoxic cardiac arrest after accidental decannulation of tracheostomy and was not the direct consequence of postoperative complications. The 1-year mortality of our population was 12% and was not associated with prolonged ICU LOS, suggesting the safety of the surgical technique and efficiency of complication treatments in critical care. The main cause of death in our study was tumor progression; the 1-year mortality in cancer patients was 14% and has been reported as high as 23% in the literature (Lahtinen et al., 2021). Contrary to our findings, large cohort studies have shown the association between complications and long-term mortality (McMahon et al., 2017; Ch’ng et al., 2014). Prognosis, as well as the risk of complication and decreased quality of life after surgery (Pierre et al., 2014), should be thoroughly discussed with the patient before obtaining informed consent for surgical intervention.

The retrospective design of our study and the size of our population were the main limits of our work. We had an accurate record of postoperative complications but some information about the medical past and preoperative investigations were not available. Therefore, the results could be influenced by a potential information bias caused by missing data. The number of deaths included in the survival analysis was also low. These limits can result in a lack of power and can explain some of the contradictory results obtained by the analysis in the primary objective. Moreover, given the number of oncological cases, we regret the lack of data on preoperative nutritional status. We defined malnutrition only on low BMI, but key elements such as hypoalbuminemia and weight loss could not be integrated (NICE guidelines, 2017). Studies have reported an association between malnutrition and poor postoperative outcomes such as complications (Shum et al., 2014; Caburet et al., 2020) and mortality (Lahtinen et al., 2021).

In 2016, the ERAS Society published guidelines (Dort et al., 2016) for optimal perioperative care of head and neck FF reconstructions. Many topics are covered in the guidelines, including postoperative pulmonary physical therapy and nutritional care. The implementation of the ERAS protocol considerably improved the evolution of postoperative care in colorectal surgery (Muller et al., 2009). The effectiveness of its implementation in head and neck FF surgeries has not yet been demonstrated (McMahon et al., 2017), but growing evidence suggests improved postoperative outcomes and reduced ICU and hospital LOS (Bater et al., 2017; Bertelsen et al., 2020; Chorath et al., 2021).

## Conclusions

Complications following maxillofacial FF surgery have a strong impact on perioperative medicine. The implementation of protocols including patient blood management and respiratory optimization could improve outcomes and should be evaluated as a part of global care improvement strategies.

## Data Availability

The datasets analyzed during this study are available from the corresponding author on reasonable request.

## Abbreviations

ASA: American Society of Anesthesiologists
BMI: Body mass index
COPD: Chronic obstructive pulmonary disease
CV: Cardiovascular
ERAS: Enhanced recovery after surgery
FF: Free flap
ICU: Intensive care unit
IV: Intravenous
LOS: Length of stay
OR: Odds ratio
PACU: Post-anesthesia care unit
